# Invariant Chain Complexes and Clusters as Platforms for MIF Signaling

**DOI:** 10.3390/cells6010006

**Published:** 2017-02-10

**Authors:** Robert Lindner

**Affiliations:** Institute of Neuroanatomy and Cell Biology, Hannover Medical School, 30625 Hannover, Germany; Lindner.Robert@mh-hannover.de; Tel.: +49-511-532-2918

**Keywords:** invariant chain, Ii, CD74, MHC II, B-cell receptor, BCR, antigen presentation, migration inhibitory factor, MIF, CD44, membrane raft

## Abstract

Invariant chain (Ii/CD74) has been identified as a surface receptor for migration inhibitory factor (MIF). Most cells that express Ii also synthesize major histocompatibility complex class II (MHC II) molecules, which depend on Ii as a chaperone and a targeting factor. The assembly of nonameric complexes consisting of one Ii trimer and three MHC II molecules (each of which is a heterodimer) has been regarded as a prerequisite for efficient delivery to the cell surface. Due to rapid endocytosis, however, only low levels of Ii-MHC II complexes are displayed on the cell surface of professional antigen presenting cells and very little free Ii trimers. The association of Ii and MHC II has been reported to block the interaction with MIF, thus questioning the role of surface Ii as a receptor for MIF on MHC II-expressing cells. Recent work offers a potential solution to this conundrum: Many Ii-complexes at the cell surface appear to be under-saturated with MHC II, leaving unoccupied Ii subunits as potential binding sites for MIF. Some of this work also sheds light on novel aspects of signal transduction by Ii-bound MIF in B-lymphocytes: membrane raft association of Ii-MHC II complexes enables MIF to target Ii-MHC II to antigen-clustered B-cell-receptors (BCR) and to foster BCR-driven signaling and intracellular trafficking.

## 1. Introduction

Invariant chain (Ii/CD74) leads at least two lives that so far remain strangely unconnected: its first life was unraveled by identifying Ii as a non-polymorphic, hence invariant, polypeptide associated with polymorphic major histocompatibility complex class II (MHC II) molecules in the late 1970s [1]. In the following 20 years, Ii has been characterized as a chaperone for MHC II that assists MHC II folding [2], prevents premature (poly-) peptide association with nascent MHC II in the endoplasmic reticulum (ER) [3,4] and targets newly synthesized MHC II to peptide loading compartments [5,6,7,8,9]. Later, Ii was also demonstrated to chaperone and target an assortment of other molecules, such as MHC I destined for cross presentation [10], CD1d [11,12], CD70 [13], angiotensin II type I receptor (ATGR1) [14] and TLR7 [15]. With the generation of Ii knockout mice, however, the first hint to a second life of Ii became available: Ii was proposed to be essential for B-cell development and was suspected to fulfill a signaling function involving the proteolytic release of its cytosolic tail peptide [16,17,18]. In 2003, an expression cloning approach revealed Ii as a receptor for the pleiotropic cytokine migration inhibitory factor (MIF) [19]. Several co-receptors that assist Ii in signal transduction have been identified in the meantime and signal transduction pathways have been assigned to MIF-Ii-coreceptor complexes [20,21,22,23,24,25,26,27,28,29]. These findings firmly establish Ii in the cytokine/signal transduction field. Here, I will provide a short overview of both lives of Ii and then briefly discuss new results that show promise to integrate the two fields. Invariant chain has been the topic of an excellent recent review by Schröder [30] and the readers are referred to this paper for a concise coverage of Ii biology.

## 2. Structure of Invariant Chain

**Figure 1:**
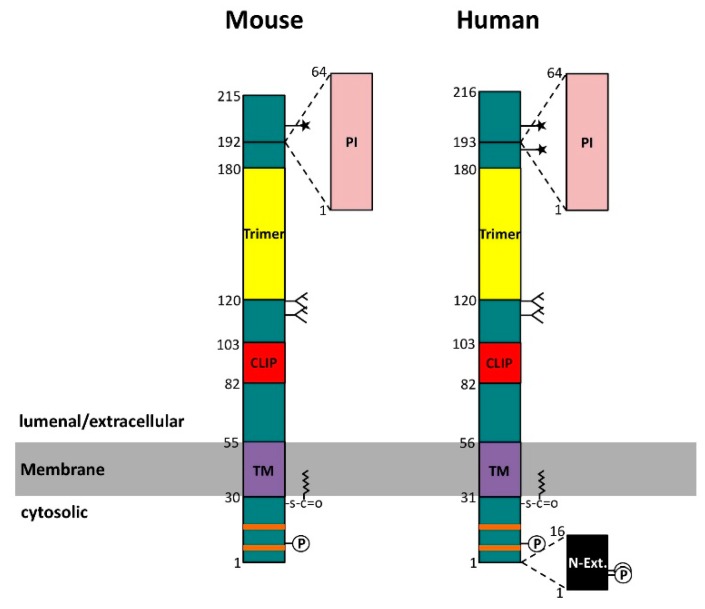
Primary structures of mouse and human invariant chain (Ii/CD74). Orange bars denote the leucine-based endosomal sorting signals; P: phosphorylation site; 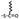: palmitoylation site; TM: transmembrane region; CLIP: class II-associated invariant chain peptide, i.e., the primary major histocompatibility complex class II (MHC II) binding segment; Trimer: trimerization domain; PI: thyroglobulin type 1 protease inhibitor domain; N-Ext.: N-terminal extension, due to alternative translation initiation; 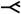: N-glycosylation site; 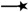: O-glycosylation site.

**Figure 2:**
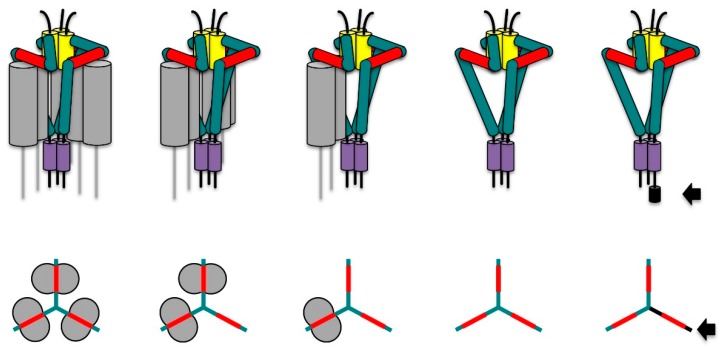
Models for invariant chain (Ii) trimers and Ii-MHC II complexes. Upper row: structural models based on the data and assumptions described in the text. Note that the main MHC II binding site on Ii is formed by the MHC class II-associated invariant chain peptide (CLIP, red segment). Secondary MHC II binding sites comprise the lateral side of the trimerization domain (yellow) and the transmembrane domain (violet). The α-chain of MHC II is oriented towards the trimerization domain of Ii as proposed by Wiley and coworkers (see text). The N-terminal extension in Ii p35 is displayed in black. Lower row: structure icons for the different association forms of Ii and MHC II as used in Figure 3. The CLIP segment is in red, the Ii p33 isoform is shown in green, the Ii p35 isoform in black (see arrow).

Ii is a type 2 transmembrane protein with a single membrane passage (Figure 1). In humans, four different isoforms have been described [31]. The short isoforms, denoted p33 and p35, differ from the long isoforms, denoted p41 and p43, by a 64 amino acid insertion encoded by the alternatively spliced exon 6. This segment is homologous to the thyroglobulin type 1 (TG-1) domain and functions as a protease inhibitor of endogenous cathepsins [32,33]. In addition to this splicing variation, an alternative initiation codon gives rise to a 16 amino acid N-terminal extension present in the p35 and the p43 isoforms of human Ii. This additional segment confers retention in the ER and will be discussed later. In mice, no such alternative initiation codon exists and therefore only two isoforms, denoted p31 and p41, are expressed. Ii forms a homotrimer [34] and this is primarily due to the lumenal region comprising amino acids 120 to 180 (Figure 1). This so-called trimerization domain is structured by three α-helices that combine with their counterparts on two other Ii chains to form a flat, cylindrical structure [35]. Trimerization of Ii is also fostered by the transmembrane segment that has been suggested to form a left-handed α-helix bundle stabilized by hydrophilic interactions in its core [36,37]. Similar to the transmembrane segment, the 29–30 amino acid long cytosolic tail of invariant chain (without the N-terminal extension) may also form a triple helical bundle [38]. Each cytosolic tail peptide in this bundle contains two leucine-based sorting motifs that are important for targeting Ii-MHC II to the endocytic pathway [39,40]. Another key region in invariant chain is a methionine-rich patch ranging from amino acid 82 to 103 (Figure 1). This so-called MHC class II associated invariant chain peptide (CLIP) region associates with the peptide-binding groove of MHC II molecules in an extended polyproline type II conformation [41,42] (Figure 2). This conformation is induced by binding to MHC II, because, in the absence of MHC II, CLIP remains unstructured [43]. The CLIP peptide is, however, not the only region of Ii that is thought to interact with MHC II molecules. In a tentative model of Ii-MHC II complexes, Wiley and coworkers proposed a further interface oriented towards the MHC II α-chain on the lateral surface of the cylindrical Ii trimerization domain [44] (Figure 2). This hypothesis could provide an explanation for the observation that the proteolytic K3 fragment of Ii, which only consists of a region C-terminal to the CLIP region, is able to bind to MHC II [45]. In addition to the K3 fragment, the Ii transmembrane segment has been identified as another site critical for the interaction of Ii and MHC II [46].

Invariant chain is post-translationally modified at several sites: on its cytosolic tail, a palmitoyl chain can be attached to a cysteine residue close to the membrane [47]. The function of this modification is not yet understood. Two serine residues in the N-terminal extension of the cytosolic tail have been shown to be phosphorylated by protein kinase C thereby affecting the transport only of p35/p43-containing Ii-MHC II complexes [48,49]. The phosphorylation of the serine residue in all the forms of human and mouse Ii by protein kinase A is important for signal transduction, as discussed later [20]. On its lumenal portion, Ii is N-glycosylated at two adjacent sites within an unstructured region located between the CLIP segment and the trimerization domain. The N-glycosides might function in protection against premature degradation [50], but this claim is yet to be substantiated in cells expressing MHC II molecules. Invariant chain is also O-glycosylated at a site C-terminal to the alternatively spliced TG-1 homology domain (Figure 1). In humans, there is an additional O-glycosylation site N-terminal to the TG-1 insertion site. A small fraction of Ii molecules carries an extensive chondroitin sulfate (CS) modification on its O-glycans [51,52]. At the cell surface, the CS form of Ii is bound to MHC II and has been shown to contact CD44 in trans (i.e., CD44 on T-cells interacts with CS-Ii-MHC II complexes on antigen presenting cells). This interaction appears to augment T-cell activation mostly likely due to the involvement of CD44 in T-cell signal transduction pathways [53].

## 3. Invariant Chain as Chaperone and Transport Co-Factor for MHC II

Ii is synthesized in the ER and trimerizes immediately after co-translational translocation [34]. Within minutes, it associates with nascent MHC II molecules by combining with preassembled MHC II α-β chains [3,54] or by sequential binding to α- and β-chains of MHC II (Neumann and Koch, 2005). By doing so, it serves as a chaperone for the proper folding of MHC II heterodimers and enhances their export from the ER and the appearance of properly folded mature MHC II molecules at the cell surface [2]. In professional antigen presenting cells, Ii is synthesized in excess of MHC II [34,54]. The surplus of Ii in the ER ensures that cytosolic peptides imported into the ER for binding to MHC I do not find empty MHC II molecules to combine with [3]. Given the excess of Ii over MHC II, one would expect that Ii trimers remain “under-saturated” with MHC II, i.e., Ii trimers should exist that are bound to only one or two MHC II heterodimers instead of three (Figure 2, middle). Contrary to this expectation, early findings by Peter Cresswell’s group provided evidence for nonameric structures in human B-cells that consisted of a trimer of Ii and three MHC II α-β heterodimers [55] (see also Figure 2, left). Work by Koch et al. contested these findings by proposing a pentameric structure composed of a human Ii trimer and a single bound MHC II heterodimer (Figure 2, middle). The authors speculated that the association of one MHC II molecule would bend the Ii trimer toward the membrane and thereby sterically exclude the binding of further MHC II molecules [56]. This publication, however, drew a lot of criticism, because the authors used a highly problematic approach to guess the molecular weight of Ii-MHC II complexes and combined this with solely negative evidence to support their model, amongst other problems (see comments accompanying the manuscript on the publishers website and [57]). More recently, an elegant functional study shone new light onto the question which type of complexes are formed between human invariant chain and MHC II [58]: the authors showed that this depended on presence or absence of the N-terminal ER retention motif in invariant chain. It had already been demonstrated earlier that the cytosolic tail of the MHC II β-chain could mask the N-terminal retention signal of Ii p35/43 and thus permitted the ER export of the complexes [59]. Now, an engineered variant of MHC II that contained an ER retention motif was expressed in HEK293 cells along with unmodified MHC II and invariant chain p33 or p35 isoforms. Only when unmodified MHC II was co-expressed with Ii p35, was export of Ii p35-containing Ii-MHC II complexes from the ER detected. No export from the ER, however, was evident when the ER-retained variant of MHC II was present in addition to unmodified MHC II and Ii p35. This suggested that invariant chain trimers of the Ii p35 isoform combined with at least two MHC II molecules, thus enabling the ER-retained MHC II variant to inhibit ER-export of unmodified MHC II bound to the same Ii-trimer. No such inhibitory effect of the ER-retained MHC II variant on the ER-export of its unmodified counterpart was detected in the presence of Ii p33 instead of Ii p35, suggesting that in this case, the lack of ER retention by Ii p33 favored the formation of pentamers containing only one MHC II per Ii trimer. In another ingenious study, Cloutier et al. refined their model by showing that Ii p35-mediated ER retention could only be relieved by an MHC II β-chain directly bound to Ii-p35 (in cis). This work provides another strong argument against the pentamer-only hypothesis proposed by Koch et al. [56], which would require MHC II to act in trans (i.e., on neighboring Ii-p35 subunits) to warrant efficient ER export of pentamers containing Ii p35 subunits [60]. Although retention in the ER appeared to be of critical importance for the type of oligomeric state of Ii-MHC II complexes in the experimental system of Cloutier et al., it may not be the only factor controlling it. In an attempt to express and purify soluble Ii-MHC II complexes devoid of cytosolic and transmembrane segments in the same type of cells (HEK293) as in the study discussed above, Majera et al. found evidence for heptamers and nonamers even in the absence of any ER-retention [61].

Once exported from the ER, Ii-MHC II complexes are transported through the Golgi apparatus and then diverted away from secretory to the endocytic pathway [5,6,39,62]. Critical for this diversion are two canonical dileucine-type of sorting motifs, which can act independently of each other [40]. These motifs serve as recognition sites for clathrin adaptor proteins [63,64], which control the formation of clathrin-coated vesicles at various membrane compartments in the cell [65]. A role in endosomal targeting of the transmembrane segment of Ii has also been invoked suggesting that additional sorting mechanisms independent of clathrin adaptors might be involved [66]. The precise route that is taken by Ii-MHC II complexes still remains a matter of debate. Whereas early findings pointed to a direct targeting of Ii-MHC II complexes from the Golgi to endocytic compartments [6,62,67], the discovery of significant amounts of Ii-MHC II complexes at the plasma membrane [68] and their rapid endocytosis [69] suggested an alternative transport route involving the plasma membrane. This model was supported by observations that the cytosolic tail of Ii interacted with the plasma membrane clathrin adaptor AP2 in vitro and that AP2 knockdown inhibited the endocytosis of Ii-MHC II complexes [8,9,63]. By contrast, other approaches ranging from cell biological transport assays and subcellular fractionation to tracking of transferrin receptor-invariant chain chimeras provided evidence for a direct intracellular delivery route confirming the original proposal [7,66].

In the endocytic pathway, Ii-MHC II complexes appear to be targeted to early endosomes first and then gradually change into more acidic and proteolytic environments as early endosomes mature to later endocytic compartments [70]. Invariant chain appears to have an active role in shaping the morphology of early endosomes and delaying the transit of the MHC II molecules through this compartment [71]. This possibly involves an interaction of the cytosolic tail of Ii with cytosolic Hsc70 [72]. Upon transit to a late endocytic compartment, Ii is progressively degraded by non-cysteine proteases and, subsequently, by cysteine proteases of the cathepsin family, mainly involving cathepsins S, L and F (reviewed in [73]). The first series of proteolytic attacks targets an unstructured region of invariant chain on the N-terminal end of the trimerization domain and leads to the severing of this domain. Subsequently, the unstructured region C-terminal of CLIP is removed by cysteine proteases from the remaining Ii, which is now termed Ii p12. At this stage, the nonameric state of Ii p12-MHC II complexes is still preserved, supporting the notion that regions outside the lumenal trimerization domain contribute to the stability of the complex [45]. A second round of proteolytical attacks by cathepsins S, L or F then targets an area N-terminal to the CLIP segment and releases the CLIP-MHC II complex from the N-terminal fragment (NTF) of invariant chain [73]. Subsequently, CLIP can be exchanged against peptides derived from exogenous or endogenous proteins degraded within late endocytic compartments. For the exchange reaction, the MHC II-like molecule leukocyte antigen DM (DM) serves as catalyst and peptide editor by transiently associating with and destabilizing CLIP-MHC II complexes (reviewed in [74]). This leads to the dissociation of the CLIP peptide. As soon as a high affinity peptide binds to MHC II, DM is displaced from the MHC II-peptide complex, which is subsequently exported to the plasma membrane on yet poorly characterized transport pathways [75]. There, it can be recognized by cognate T-cell receptors and provide a critical signal for the initiation of an adaptive immune response. 

## 4. Invariant Chain as Receptor for Migration Inhibitory Factor (MIF)

MIF is a small secretory protein with a molecular weight of 12.5 kDa that has been shown to form homotrimers in solution [76]. Its primary structure does not contain a signal sequence [77] and MIF is secreted on an unconventional pathway involving ABCA1 [78]. Many types of immune and non-immune cells have been described to produce MIF and its secretion has been shown to be up-regulated by exposure to pathogen-associated molecular patterns [79]. MIF has been one of the first cytokines to be identified and since then, a pleiotropic assortment of functions ranging from chemotaxis to proinflammatory and cell survival effects have been ascribed to it (reviewed by [80,81]). It is therefore no surprise that MIF is involved in a wide variety of pathologic conditions such as atherosclerosis, sepsis, autoimmunity and tumor survival just to name a few. The interested reader is referred to the extensive medical literature on this subject. MIF also shows a tautomerase activity in vitro, whose in vivo significance is unclear at present. A close homologue, dopachrome tautomerase (D-DT, now called MIF 2) has been demonstrated to account for residual MIF-like cytokine activity in MIF knockout mice [82].

In 2003, invariant chain was identified by an expression cloning approach as the first receptor for MIF [19]. Truncation experiments with invariant chain implicated the region C-terminal to CLIP segment (amino acids 109–149) to be critical for MIF binding. Evidence from experiments with a proteolytic fragment of Ii (K3 comprising amino acids 110 to ~180) [45] and a tentative model derived from an NMR approach [44] suggested that this region of Ii may form an MHC II interaction surface secondary to the main binding site CLIP. Indeed, peptide-loaded recombinant fragments of MHC II consisting of a linked α1 and β1 domain (RTL = recombinant T-cell ligand) have recently been shown to compete with MIF for binding to a similar surface region of invariant chain [83,84]. Using computer-based modeling, these authors presented further evidence in support of their hypothesis and proposed two hexapeptide stretches on Ii that might represent shared binding sites of MIF and MHC II [85]. It remains to be seen, however, to what extent the interactions of the recombinant MHC II fragments resemble the behavior of intact MHC II molecules and how the presence of MHC II α2 and β2 domains and the sterical constraints imposed by the membrane anchorage affect the interactions proposed in these studies.

**Figure 3:**
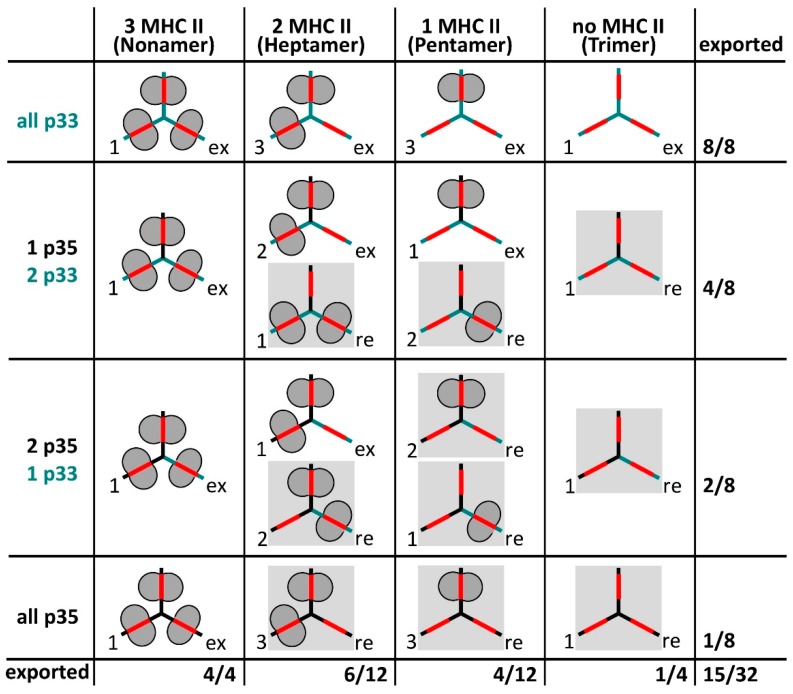
Combination matrix for different association forms of Ii p33, Ii p35 and MHC II and their endoplasmic reticulum (ER) retention phenotype, based on results from Thibodeau and coworkers (see text). The number of different association forms in respect to MHC II binding is given to the left of each icon, the retention phenotype to the right (re = retained in the ER, ex = exported to endosomes and the cell surface). The fraction of exported association forms is given to the right and at the bottom of the matrix. Note that from a total of 32 association forms, 15 should be exported to endosomes and the cell surface.

Most studies on MIF binding to Ii have been performed in heterologous overexpression systems in the absence of MHC II. To assess the binding of MIF to Ii on professional antigen presenting cells, the actual amount of free Ii trimers at the cell surface and a potential binding to sub-stoichiometric Ii-MHC II complexes has to be considered. Human professional antigen presenting cells express about 20% of Ii with the N-terminal extension as p35 or p43 isoforms [57]. Therefore, a large fraction of all Ii-trimers should be expected to contain at least one N-terminal retention motif. Different isoforms are thought to be statistically incorporated into Ii trimers [34] and therefore a variety of different Ii-MHC II complexes can be envisioned to be present in the ER (Figure 3). These complexes should be differentially retained in the ER depending on whether they display a yet unmasked ER retention motif on Ii or not. Complexes that still contain unmasked retention motifs (in grey boxes in Figure 3) would be only released from the ER after binding of additional MHC II, i.e., after conversion to heptamers or nonamers. Importantly, a subset of Ii-MHC II complexes should be able to leave the ER, although these complexes are not yet saturated with MHC II. Given the ratio of p35/p43 to p33/p41, only few free Ii-trimers without a retention signal are supposedly formed. Therefore, only a marginal number of free Ii-trimers would be expected to leave the ER, even in the presence of a large excess of Ii over MHC II. This small fraction should be heavily outnumbered by Ii trimers bound to one or two MHC II molecules (i.e., pentamers and heptamers) that are able to exit the ER due to masking of all retention motifs by MHC II (Figure 3). For these reasons, most surface Ii in human professional antigen presenting cells should be sub-stoichiometrically complexed with MHC II and only very little free Ii trimers should be present at this location.

In mouse professional antigen presenting cells, the absence of any known ER retention motif and the synthesis of excess Ii over MHC II might be expected to give rise to a surplus of free Ii over supposedly mostly pentameric Ii-MHC II complexes at the cell surface. However, evidence from sedimentation analyses with lysates of a mouse B-cell line partially refutes this hypothesis [86]: only 3% of surface Ii sedimented at the position of free trimers, whereas 97% of surface Ii was associated with MHC II distributing to two distinct peaks, which tentatively represented pentameric and heptameric Ii-MHC II complexes. The amount of Ii-bound MHC II β-chain at the cell surface of M12.C3F6 cells is estimated to be ~0.01% of total surface MHC II β-chain (R. Lindner, unpublished). Because the number of cell surface MHC II molecules in M12.C3.F6 has been determined previously (~2 × 10^6^ per cell [87]), about 200 Ii-MHC II complexes should be expected to reside temporarily at the cell surface. This would limit the number of free Ii trimers at the plasma membrane of M12.C3.F6 cells to ~6 molecules per cell at steady state. In the face of such low levels of free Ii, it appears likely that sub-stoichiometric Ii-MHC II complexes serve as additional MIF receptors, given the Ii-dependent sensitivity of B-cells towards MIF [26,29,88].

At the time when invariant chain was identified as a receptor for MIF, a signaling function of Ii had only been considered in respect to B-cell development, possibly involving intra-membrane proteolysis and release of the intracellular domain (ICD) fragment as a putative signal transducer (see below). For MIF, however, some signal transduction events had already been described, but it remained unresolved, how they might be triggered by Ii. The situation dramatically changed with the description of CD44 as a signal transducing co-receptor of Ii [20,89] (see below). This finding was surprising because CD44 had originally been reported to interact with Ii in trans, i.e., with Ii and CD44 located in the plasma membranes of B and T-cells, respectively [53]. By contrast, the co-receptor function described in the above-mentioned studies was based on an interaction in cis. Furthermore, Naujokas et al. showed that CD44 interacted with the chondroitin sulfate-modified isoform of Ii, whereas Shi et al. [20] only presented data on Ii p31 (lacking this modification). Evidently, the cis and the trans effects of CD44 do not bear any relationship despite having often been cited as interdependent. Nevertheless, CD44 is known to activate tyrosine phosphorylation [90] and the availability of transfection systems based on Ii/CD74- and CD44-negative cell lines and of knockout mice [20,29] unequivocally demonstrated a cooperation (in cis) between Ii/CD74 and CD44 in transmitting MIF-generated signals.

In addition to Ii/CD74, three G protein-coupled receptors of the CXC chemokine family have been identified as receptors for MIF: CXCR2, CXCR4 and, most recently, CXCR7, which has also been termed atypical chemokine receptor 3 (ACKR-3) [23,25,26,27,91,92]. Although MIF is not related to chemokines, it has been shown to contain sites that are similar to the N-terminal region and the N-loop segment of chemokines. These are required to interact with two distinct sites on the chemokine receptors, however, the precise segments of MIF engaged in interactions with CXCR2 and CXCR4 appear to differ. Furthermore, the binding site 2 on the chemokine receptors includes a transmembrane cavity that is occupied by the unstructured N-terminal regions of chemokines in the course of binding, a process important for receptor activation. With MIF, however, no such interactions have been detected, suggesting a mechanism of receptor activation that differs from chemokines [92]. The chemokine receptors may not only compete with Ii in binding of MIF, but they may also function as co-receptors for Ii because CXCR2 and CXCR4 have been shown to form complexes with Ii/CD74 by co-immunoprecipitation [23,25]. It is not known at present, however, whether the chemokine receptors bound directly to Ii/CD74 or whether these complexes also contained CD44.

## 5. MIF-Induced Signaling from Invariant Chain Complexes

The first hints to a signaling function of Ii were revealed with the discovery of a defect in B-cell development in Ii-knockout mice [16]. B-cells in Ii^−/−^ mice showed an immature phenotype, which was not present in MHC II Aβ^−/−^ mice, suggesting that the defect in B-cell development in Ii^−/−^ mice might be independent of the MHC II-directed functions of Ii. In an attempt to confirm this hypothesis, Shachar and coworkers used a transgenic mouse that expressed an N-terminal fragment of Ii (1-82) under a B-cell specific promoter on an Ii^−/−^ background. The Ii 1-82 fragment did not support the formation of stable, peptide-loaded MHC II molecules and their transport to the cell surface, but it relieved the B-cell developmental block, suggesting that the N-terminal part of Ii was sufficient to drive this process [93]. This model of Ii-driven B-cell development remains controversial, however, because of contradictory data on the MHC II dependence: It has been reported that the expression of unpaired MHC II β-chain can have adverse effects on the survival of B-cells [94]. To rule out this potential issue, Matza et al. used double knock-out (KO) mice negative for Ii and MHC Aβ-chain [93]. The B-cells of the double KO mice showed a similar immature phenotype as the B-cells of Ii^−/−^ mice suggesting that the developmental block was not caused by the presence of potentially toxic, MHC II β-chains. By contrast, in a later study using double KO mice devoid of Ii and all MHC II subunits (including the Eβ-chains), normal B-cell development was observed [95]. Therefore, it was concluded that the B-cell development block was caused by a problem with MHC II in the absence of Ii rather than by the lack of positive signal transmitted by Ii. The Ii 1-82 fragment used by the Shachar group [18,93] may have alleviated the problem with MHC II by chaperoning these molecules using its transmembrane region. In fact, complexes between MHC II and short, CLIP-less fragments of Ii have been demonstrated in transfected cells upon extraction with mild detergent [46].

### 5.1. The RIP Pathway

**Figure 4:**
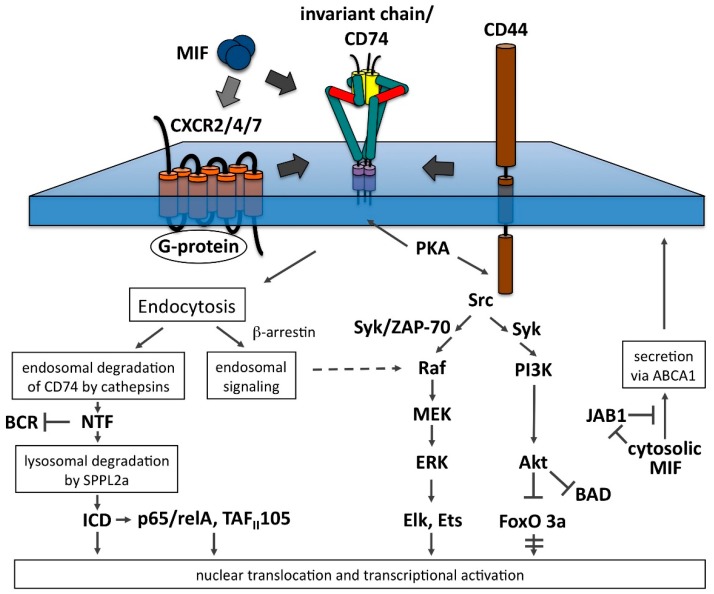
Signal transduction pathways emanating from migration inhibitory factor (MIF)-bound invariant chain-CD44 complexes. Only components activated upon MIF treatment are listed in the scheme. Note that the scheme does not account for signal transduction pathways initiated by CXCR-type chemokine receptors, although some of the signaling molecules shown here may also be targeted by these receptors. CXCR 2, 4, and 7 have been shown to bind MIF and to associate with Ii/CD74. They may work as signal transducing co-receptors for Ii/CD74 in some cases (see text).

Work by Dobberstein, Shachar and colleagues has shed light on the fate of Ii/CD74-NTF [18,96,97]. It is cleared from the membrane by regulated intramembrane proteolysis (RIP), which causes the two resulting fragments to be released to either side of the membrane. This process resembles the way in which the intra-membrane proteases presenillin and signal peptidase act on their substrates [30]. The protease in charge of cleaving NTF within the membrane has recently been identified as a member of the signal peptidase family and has been termed signal peptide peptidase-like 2a (SPPL-2a) [98,99,100,101]. Unlike signal peptidase, SPPL-2a is located in late endosomes and lysosomes suggesting that the intramembrane cleavage of Ii-NTF may play a role in the final catabolism of Ii. Studies based on SPPL-2a-deficient mice, which are not able to process NTF to ICD, have revealed a severe defect in B-cell development [98,99,100,101]. This defect was accompanied by reduced BAFF-R surface expression, altered B-cell receptor (BCR) trafficking and reduced tonic BCR signaling. These negative effects appear to have been caused by an association of accumulated NTF with BCR [102]: When SPPL-2a^−/−^ mice were crossed with CD74^−/−^ mice, the severe defect in B-cell development was reversed to a level seen in CD74^−/−^ mice, suggesting that the severe B-cell developmental defect in SPPL-2a^−/−^ mice was indeed caused by a negative effect of NTF (Figure 4).

There is evidence, however, that Ii also assumes a positive function in signaling via its intracellular domain (ICD), which is released by regulated intramembrane proteolysis (RIP) of NTF. Such a function is exemplified by sterol regulatory element binding protein, whose ICD is released in the Golgi complex and subsequently is imported into the nucleus to serve as transcription factor [103]. Using heterologous and B-cell-based overexpression systems, the ICD fragment of invariant chain (Ii1-42) was demonstrated to be released into the cytosol by regulated intramembrane proteolysis (RIP) and to be imported into the nucleus. There, it activated a transcription program relying on the NFκB p65/RelA homodimer and its co-activator TAF_II_105 [17,18,93,97] (Figure 4). This process was shown to lead to the generation of a number of downstream activators, including Bcl-X_L_ [24], TAp63 [104], Bcl-2 [104] and IL-8 [88] that promoted B-cell survival.

With the discovery of Ii as a receptor for MIF and CD44 as a signal transducing co-receptor, the link between MIF-triggered signaling and the proposed ICD-dependent positive signaling function has been investigated [29]. MIF induced an upregulation of ICD in primary B-cells that was dependent on Ii/CD74 and CD44, however, the magnitude of the effect was only moderate. Nevertheless, the subsequent up-regulation of mRNA levels of candidate genes such as Bcl-2, Bcl-X_L_ or cyclin E was detected and its slow time course would be in agreement with the expected delay in ICD release from slowly accessed late endocytic compartments. 

Due to its small size and its tendency to be degraded by the proteasome [105], the ICD fragment was proposed to associate with other proteins, such as transcription factors, to be able to exert transcriptional regulation. Although a requirement for the NFκB p65/RelA homodimer for B-cell maturation was demonstrated early-on [17], only very recently an interaction between ICD and the transcription factors Runx1,3 and RelA, but not RelB, has been shown by pulldown from human B lymphoma cells [106]. The authors were also able to detect ICD-RelA complexes in fractions derived from the cytosol and the nucleus, suggesting that ICD bound to Rel A in the cytoplasm prior to the import of the complex into the nucleus. Nuclear translocation of ICD, as demonstrated earlier by Becker-Herman et al. [97], has also been confirmed in a recent study employing a β-galactosidase complementation assay [105]. These authors also investigated a transcriptional role of the ICD fragment using a 293T cell-based expression system in which they could trigger the release of plasma membrane-targeted ICD via tobacco etch virus nuclear inclusion-a (TEV) protease. Subsequent microarray and real-time PCR analyses revealed the upregulation of mRNA coding for secreted frizzled-like protein 2 (SFRP-2) in the 293T cells and its downregulation in bone marrow-derived dendritic cells (bmDCs) from Ii/CD74^−/−^ or SPPL2a^−/−^ mice. SFRP-2 has been described as a wnt-regulatory protein secreted in multiple myeloma to suppress osteoblasts [107], but its role in bmDCs still needs to be investigated. Surprisingly, no upregulation of NFkB-regulated genes has been detected in this study, an effect well established for ICD in the 293T cell line as well as in primary B-cells [17,24,29,104]. The latter observations have been recently confirmed and largely extended in the context of the B-cell malignancy chronic lymphocytic leukemia [106]. The authors analyzed the chromatin binding sites of ICD and its effects on transcription by a low bias approach involving chromatin immunoprecipitation sequencing and RNA sequencing. They identified consensus binding motifs associated with the Runx- and NFκB transcription factor families, showed their interaction with ICD by pulldown and systematically characterized transcription processes mediated by these factors upon activation of Ii/CD74 in B-lymphoma cells derived from patients. Although their initial experiments were based on the same 293T cell line used by Mentrup et al. [105], they do not comment on the discrepancies between the two studies.

### 5.2. The PI3K-Akt Pathway

This pathway is triggered by the activation of phosphatidylinositol 3-kinase (PI3K) that generates phosphatidylinositol (3,4,5)-trisphosphate (PIP_3_) on the cytosolic leaflet of the plasma membrane. The pleckstrin homology (PH) domain of Akt is specific for PIP_3_ and mediates the recruitment to the plasma membrane, where Akt is activated by dual phosphorylation at Thr308 and Ser473. In addition to phosphorylating numerous central regulators of cell growth and cell cycle progression, such as mTor or GSK-3, it also inactivates the pro-apoptotic protein Bad and FoxO transcription factors. Its role in cell survival is furthermore documented by its activation of MDM2, which targets p53 for degradation [108]. Binding of MIF to Ii/CD74-CD44 complexes has been demonstrated to trigger rapid and transient phosphorylation of Akt [20,22,29,109] (Figure 4). Although a requirement for PI3K in MIF-induced Akt activation has been demonstrated as well [22,109], it is still unclear, how PI3K is activated. A Src kinase is suspected to be involved, because phosphorylation of Akt was found to be inhibited by the Src kinase inhibitor PP2 and in Src^-/-^/Yes^-/-^/Fyn^−/−^ cells [22,109]. Furthermore, Src kinase was phosphorylated upon MIF stimulation in primary B-cells and this effect was dependent on the presence of Ii/CD74 and CD44 [29]. In T-lymphocytes, CD44 has been shown to be associated with the Src tyrosine kinase Lck that was activated upon receptor oligomerization [90]. A similar association/activation may occur in other cell types and might transmit the signal from Ii-bound MIF to the PI3K/Akt pathway, although this has not been formally shown yet. MIF-induced activation of the PI3K/Akt pathway resulted in the induction of anti-apoptotic factors (Bcl-2 and Bcl-X_L_), cell cycle regulators (Cyclin E) and NFκB activity in primary B-cells [29] and inhibitory phosphorylation of Bad, Foxo3a and GSK-3 in transformed fibroblasts [22]. The activation of the PI3K/Akt pathway with its cell survival promoting effects by the MIF-CD74/CD44 axis is congruent with observations that various tumors express Ii/CD74 and MIF shows a tumor promoting effect in some systems [30].

The PI3K/Akt pathway appears to be interacting with the RIP pathway: inhibitors of Syk and of PI3K have been shown to decrease the formation of the ICD fragment stimulated by treating primary B-cells with a polyclonal antibody against Ii/CD74 [24]. Because MIF activated the Akt/PI3K pathway with similar kinetics and also enhanced the release of the ICD fragment at the same scale [29], it appears reasonable to assume that MIF augments the formation of the ICD fragment via the PI3K/Akt pathway. It remains to be seen, however, at which precise step the PI3K/Akt pathway exerts its effect on the RIP pathway.

### 5.3. The MAPK/ERK Pathway

This pathway is activated by many receptors involved in growth and differentiation and there are numerous variations on the level of individual components. The basic architecture of the pathway includes a receptor, an adaptor that couples to a guanine nucleotide exchange factor, which in turn transmits the signal to a small membrane-bound GTPase such as Ras. Its GTP-bound form is recognized by the first member of the core kinase cascade, Raf, which passes the signal on by phosphorylating and activating MEK1,2 kinase, which in turn phosphorylates and activates the MAPkinase ERK1,2. Activated ERK can translocate into the nucleus and phosphorylate a large set of transcription factors including Elk and Ets (reviewed in [108,110]). MIF-induced phosphorylation of ERK1,2 followed by the activation of phospholipase A2 and the release of arachidonic acid has been one of the first reported molecular mechanisms behind the action of MIF [111]. This effect was dependent on the production and secretion of endogenous MIF by the cells (autocrine activation loop) and gave rise to a sustained activation of ERK (Figure 4). Interestingly, exogenous MIF may contribute to the endogenous MIF level after internalization by its surface receptor [112], although the details of its translocation into the cytosol have not been clarified yet. The sustained type of ERK activation has been shown to be positively and negatively regulated by JAB1/CSN5 [21], a COP9 signalosome component present in the cytosol, which is bound by cytosolic MIF. The precise mechanism of this interference remains to be elucidated. Because JAB1 is involved in the regulation of AP1-dependent transcription and the cell cycle, and MIF inactivates JAB1 by binding to it, the cytosolic pool of MIF appears to be engaged in an additional regulatory circle distinct from the MAPK/ERK pathway. Two additional pathways for the sustained activation of ERK have been proposed: one involves the small GTPase Rho, myosin light chain kinase, focal adhesion kinase and ERK in adherent fibroblasts, which appear to maintain autocrine secretion of MIF in response to adhesion and stress fiber formation [113]. The other pathway implicates β-arrestin 1 in the internalization of MIF-Ii/CD74 complexes into endosomes from where sustained activation of the ERK pathway is proposed to arise [114]. Since arrestins are recognized as important signal transduction modules for G protein-coupled receptors [115], this work will surely trigger a closer scrutiny of the proposed activation mechanism.

By contrast, the rapid and transient activation of ERK was independent of the autocrine activation loop and involved the phosphorylation and activation of a number of key mediators of the MAPK/ERK pathway, comprising Raf-1, MEK, ERK and Elk-1 [20,21] (Figure 4). In addition, a role for Src kinase in upstream signaling was demonstrated by using inhibitors and Src deficient cells. Furthermore, phosphorylation of the cytosolic tails of Ii/CD74 and CD44 by protein kinase A (PKA) in response to MIF binding has been reported, extending earlier information on the essential role of this kinase in MIF-induced ERK activation [111]. The precise function of these modifications and the way by which PKA was activated by MIF remain to be clarified. Recently, a critical involvement of ZAP-70 in MIF-dependent B-cell migration and signaling to the MAPK/ERK pathway has been demonstrated. Interestingly, antibody-mediated blockade of Ii/CD74, or chemical inhibition of CXCR4 abrogated MIF-triggered chemotaxis in these cells, suggesting that the two receptors cooperated [26].

## 6. Ligand-Induced Signal Cluster Formation Enabled by Membrane Rafts

**Figure 5:**
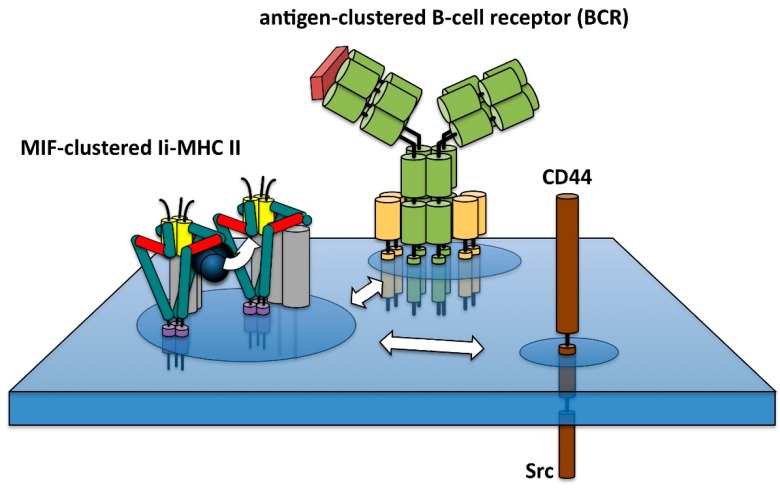
Hypothetical model for the ligand-induced formation of raft-dependent signaling clusters. Note that the proposed lateral binding of MIF to Ii-MHC II complexes may trigger direct clustering of Ii-MHC II complexes at suitable input ratios of MIF and Ii-MHC II, whereas an excess of MIF should be expected to inhibit this reaction. Subsequent co-clustering and signal transduction should be affected accordingly. Also note that the interaction of Ii/CD74 and CD44 most likely depends on protein–protein interactions in addition to the weaker lipid-mediated association.

Ii-MHC II complexes have been shown to distribute to membrane rafts [116,117,118]. These are defined as small, dynamic, heterogeneous, cholesterol- and sphingolipid-rich membrane patches that function in the compartmentalization of cellular processes [119]. They can be stabilized to form larger-sized and longer-lived membrane domains by clustering of their components [120]. Clustering can be induced by ligands and the B-cell receptor is a paradigm for such a (transient) stabilization [121]. In a recent paper, Julian Hauser and I have demonstrated that the independent clustering of Ii-MHC II complexes and the BCR triggered their coalescence into a membrane raft [86]. The induced coalescence was found to enhance BCR-driven signaling and to co-target both protein complexes to endosomes. This synchronized delivery of BCR-bound antigen and newly synthesized MHC II to endocytic compartments may foster antigen presentation. We furthermore showed that co-clustering of invariant chain with the BCR could also be triggered by its natural ligand MIF, however, under our conditions, this required both, BCR and Ii-bound MIF, to be clustered by polyclonal antibodies. Here, two scenarios are proposed, in which the clustering of the BCR and Ii/CD74 could occur in vivo: (A) Clustering of the B-cell receptor at the cell surface has been induced by oligomerized antigen or antigen containing multiple epitopes for the BCR [122]. However, the need for oligomerized antigen could be bypassed by monomeric antigen tethered to cellular membranes, which induced transient BCR clustering and subsequent signal transduction events [123,124]. Such a membrane tethering also occurred upon capture of antigens by monocytes, dendritic cells and follicular dendritic cells, which were involved in antigen delivery to B-lymphocytes in vivo [125,126,127]. Similarly, it has been shown that MIF can be displayed in a cell-bound form to other MIF-receptor-bearing cells [25], suggesting that it might trigger transient clustering of its receptor Ii/CD74 similar to what was observed for antigen and BCR [123,124]. A hypothetical model involving membrane-tethered antigen and MIF has been proposed by us in an earlier work [86]. (B) New modeling data on how trimeric MIF interacts with the lateral side of invariant chain C-terminal to the CLIP segment [83,84,85] now opens the possibility that the two other subunits of trimeric MIF interact with more Ii-MHC II complexes, if there are no steric restrictions (Figure 5). If this condition is met, the additional interactions should lead to the clustering of Ii-MHC II complexes. The size of the clusters should depend on the ratio of MIF to Ii-MHC II-complexes and on the type of Ii-MHC II complexes available on the cell surface. Sufficient clustering of Ii-MHC II by MIF is expected to generate a stabilized membrane raft that would allow the coalescence with the antigen-clustered B-cell receptor on the basis of mixing similar, lipid raft-type membrane environments. The generation of such an membrane raft-enabled platform should foster the initiation of signal transduction processes as observed with model receptors such as the BCR [128]. It may be rewarding to also include molecules that function as co-receptors (CD44, CXCR2, CXCR4 and CXCR7) in the consideration of co-clustering receptors of MIF, because some of them appear to be associated with membrane rafts as well [129,130,131] and this might further support the formation of membrane raft-enabled signaling platforms.

## 7. Conclusions

Professional antigen presenting cells express only very few free Ii/CD74 trimers at the cell surface that can serve as receptors for the cytokine MIF. Instead, a considerable number of MHC II-under-saturated Ii complexes is present at this location in the form of pentamers (Ii_3_(ab)) and heptamers (Ii_3_(ab)_2_). These complexes contain one or two free invariant chain subunits that may bind MIF. MHC II-under-saturated Ii complexes may therefore be the prevalent MIF receptor on professional antigen presenting cells. Ii-MHC II complexes show a strong association with membrane rafts and can—in a clustered state—form long-lived membrane domains capable of coalescing with other membrane raft domains, such as the one occupied by antigen-clustered BCR. It will be important to determine which forms of surface Ii/CD74 interact with MIF and how this affects its association with signal transducing co-receptors such as CD44 or CX chemokine receptors and MIF-induced signaling in general. Moreover, the capability of Ii-MHC II complexes to form signaling platforms with other raft-preferring receptors such as the BCR has to be taken into consideration in the analysis of MIF-mediated signal transduction.
